# Metabolic Syndrome Prevalence among Northern Mexican Adult Population

**DOI:** 10.1371/journal.pone.0105581

**Published:** 2014-08-20

**Authors:** Rogelio Salas, Maria del Mar Bibiloni, Esteban Ramos, Jesús Z. Villarreal, Antoni Pons, Josep A. Tur, Antoni Sureda

**Affiliations:** 1 Faculty of Public Health Nutrition, Autonomous University of Nuevo León, Monterrey, Mexico; 2 Research Group on Community Nutrition and Oxidative Stress, University of Balearic Islands and CIBERobn (Physiopathology of Obesity and Nutrition), Palma de Mallorca, Spain; 3 Department of Health of the State of Nuevo León, Monterrey, Mexico; Children’s National Medical Center, Washington, United States of America

## Abstract

**Background and Aims:**

Dietary habits in the Mexican population have changed dramatically over the last few years, which are reflected in increased overweight and obesity prevalence. The aim was to examine the prevalence of metabolic syndrome (MetS) and associated risk factors in Northern Mexican adults aged ≥16 years.

**Methods and Results:**

The study was a population-based cross-sectional nutritional survey carried out in the State of Nuevo León, Mexico. The study included a sub-sample of 1,200 subjects aged 16 and over who took part in the State Survey of Nutrition and Health–Nuevo León 2011/2012. Anthropometric measurements, physical activity, blood pressure and fasting blood tests for biochemical analysis were obtained from all subjects. The prevalence of MetS in Mexican adults aged ≥16 years was 54.8%, reaching 73.8% in obese subjects. This prevalence was higher in women (60.4%) than in men (48.9%) and increased with age in both genders. Multivariate analyses showed no evident relation between MetS components and the level of physical activity.

**Conclusions:**

Obese adults, mainly women, are particularly at risk of developing MetS, with the associated implications for their health. The increasing prevalence of MetS highlights the need for developing strategies for its early detection and prevention.

## Introduction

Metabolic syndrome (MetS) is a disorder characterised by the presence of multiple risk factors, including central obesity, hyperglycaemia, hypertriglyceridemia, low plasma HDL-cholesterol and hypertension. Concurrence of at least 3 of these factors means that an individual has MetS [1]. The relation of MetS with the risk of developing several chronic diseases, such as diabetes and cardiovascular diseases (CVD), is well established, and it is also associated with a high mortality risk [2]. The aetiology of MetS, although largely unknown, is considered to reside in a complex interaction between genetic predisposition and metabolic and environmental factors [3].

In developed countries MetS appears to affect around 25% of the population^4^. Moreover, its prevalence is increasing rapidly throughout the world, in parallel with the increasing prevalence of diabetes and obesity, and becoming a major public health problem [4]. In fact, in Latin America, obesity affects both sexes and all ages, it is reported to increase with age and is more prevalent in women [5]. Obesity, especially abdomino-visceral, is associated with pathogenic factors that contribute to an increase in chronic non communicable diseases such as CVD, cancer and MetS [5], [6]. Using the MetS definition by the National Cholesterol Education Program Adult Treatment Panel III (ATP III), the prevalence of MetS in Mexican adults was 26.6% in 1993 [7], the ENSANUT 2006 project reported that the prevalence of MetS had gone up to 36.8% [8], and the last Mexico nutritional survey (ENSANUT 2012) reported that MetS had increased to 45% [9]. Accordingly, cardiovascular disease is the primary cause of death for both sexes in Mexico [10]. This increased tendency could be associated with significant changes in lifestyle behaviour including physical inactivity, high carbohydrate diets, alcohol, and tobacco consumption [11].

It is well established that regular physical activity prevents both the incidence of chronic diseases and premature death [10]. Many studies reported that regular exercise confers health benefits by reducing cardiovascular risk factors, including elevated blood glucose and triglyceride levels, low HDL-cholesterol and increased waist circumference [10]. All together is associated with a reduced risk of type 2 diabetes [12] and MetS [13].

In the present study we aimed to assess the prevalence of MetS among the State of Nuevo León (Mexico) adult population and its association with physical activity. The association between age, gender and the MetS was also investigated.

## Methods

### Study design

The study was a population-based cross-sectional nutritional survey carried out in the State of Nuevo León, Mexico (2011–2012).

### Study population, recruitment and approval

This study is part of the State Survey of Nutrition and Health–Nuevo León 2011/2012 (EESN-NL 2011/2012). The EESN-NL 2011/2012 was designed by the National Institute of Statistics and Geography (INEGI) to obtain information on the health and nutritional status of the population living in the State of Nuevo León. The state was divided into four regions: northern, central, southern and the Metropolitan area. Neighbourhood blocks were randomly selected and all subjects in all households were invited to be surveyed. A target of 1059 households per region was estimated using the household as the sampling unit and an average of 2 interviews per household. The sample size was considered to be large enough to detect risk factors at regional level, that have, at least, a prevalence of 8%, with a relative error calculation of 15% and a non-response rate of 40%. This sample size also calculated a prevalence of 9.0% in individuals aged 0 to 9 years, 10.0% in those aged 10 to 19 years, 5.0% in those aged 20 to 59 years, and 17% of the population aged 60 years and over. Information was obtained from 4,236 households and 7,290 individuals (0–9 years-old: 1,372; 10–19 years-old: 1,319; 20–59 years old: 3,125; ≥60 years-old: 1,474). No two participants in the same age group per household were selected.

### Sample selection

A sample size of 640 households was considered sufficient to detect risk factors with 95% confidence and a precision rate of 10%. This analysis was limited to adult participants aged 16 years and over who provided a fasting blood test, with no missing data, needed to calculate their MetS risk. Statistical software was used to select, at random, half the study participants to be women (54% from the 16–25 year old group; 37% from the 26–45 year old group; 42% from the 46–65 year old group; and 82% from from the over 65 year old group), leaving an analysis sample of 1,200 people (51.1% women). Data on physical activity was available for 977 participants (51.8% women). The study was conducted according to the guidelines laid down in the Declaration of Helsinki, and all procedures involving human subjects were approved by the Scientific Technical Committee of the Public Health and Nutrition Faculty at the Autonomous University of Nuevo León. Written informed consent was obtained from all subjects and also from the next of kin, carers, or guardians of the minors involved in the study.

### Anthropometric measurements

Height was determined to the nearest millimetre using a mobile stadiometer (SECA 213, Birmingham, United Kingdom), with the subject’s head in the Frankfurt plane. Body weight was determined to the nearest 100 g using a digital scale (Seca 813, Hamburg, Germany). Waist circumference (WC) and hip circumference (HC) were measured to the nearest 0.1 cm using a non-stretch measuring tape (Gulik, Lafayette Instrument Co, IN, USA). Body mass index (BMI) and waist-to-height ratio (WHtR) were also calculated.

Manual blood pressure (BP) measuring instruments and accessories (Hergom, Beijing Hergom International Business Co, Beijing, China) were used and BP measurements were performed to the nearest 1 mmHg and were taken from seated participants with the right arm resting and palm facing upward. Two readings were taken 5 min apart, and the average of the two readings was taken. If the difference between the first and the second reading was ≥10 mmHg for systolic pressure (SBP) and/or ≥6 mmHg for diastolic pressure (DBP), then a third measurement was made, and the average of all three measurements was calculated.

### Biochemical assays

Venous blood samples were obtained from the antecubital vein in suitable vacutainers after 12-hr overnight fasting conditions. Blood samples were centrifuged at 900×g at 4°C for 15 min and the supernatant was recovered and used to measure biochemical parameters. Serum glycaemia, triglycerides (TGs), total cholesterol, HDL-cholesterol, LDL-cholesterol, and VLDL-cholesterol were determined using the Cobas 6000 analyser series (Roche), by the Metropolitan Hospital “Dr. Bernardo Sepúlveda” laboratory’s of the Ministry of Health of Nuevo León, Mexico.

### Metabolic syndrome definition

MetS was defined according to the census definition (IDF/NHLBI/AHA/WHF/IAS/IASO) [14]. Participants were defined as having MetS if they met, or exceeded, the criteria for three or more of the following five variables: a) WC ≥90 cm in men and ≥80 cm in women; b) serum triglyceride ≥150 mg/dL; c) HDL-cholesterol <40 mg/dL in men and <50 mg/dL in women; d) BP≥130/85 mmHg; and e) fasting serum glucose level ≥100 mg/dL. Treatment with diabetic or blood pressure medication was considered as a positive variable.

### Physical activity questionnarie

The International Physical Activity Questionnaire (IPAQ) [15] short form was used to assess participants’ physical activity during the previous 7 days by the frequency (d/wk), duration (min/d or h/d), and intensity (sedentary, light, moderate, or vigorous) of physical activity. According to the IPAQ scoring protocol, responses were converted to Metabolic Equivalent Task minutes per week (MET-min/wk): total minutes over last 7 days spent on light, moderate, and vigorous activity were multiplied by 3.3, 4.0, and 8.0, respectively, to create MET scores for each activity level. Physical activity levels were also classified into three categories: low, moderate and high, according to the scoring system provided by IPAQ.

### Statistical analysis

Analyses were performed with the SPSS statistical software package version 21.0 (SPSS Inc., Chicago, IL, USA). Almost all tests were stratified by gender. Significant differences between group prevalence rates were calculated by means of χ^2^ test and the two-tailed Fisher’s exact test when the expected frequency in any cell was less than 5. Differences between group means were tested by unpaired *t*-test or an ANCOVA adjusted by age. Univariate analysis of the total sample (logistic regression analysis considering the effect of one explanatory variable) was used to assess the association between age (independent variable) and each of the MetS component (dependent variables). Multivariate analyses (multiple logistic regressions considering the simultaneous effect of each explanatory variable adjusted for age and gender) were used to assess the association between physical activity level (independent variable) and each of the MetS components (dependent variables).

## Results

Table 1 shows the characteristics of the participants. No significant differences between men and women were reported in age, fasting glycaemia level, treatment for diabetes, LDL-cholesterol, VLDL-cholesterol, total cholesterol, TGs, SBP and DBP and treatment for hypertension. There were significant differences in weight, height, BMI, WC, WHtR, HDL-cholesterol and physical activity levels between men and women. Men were significantly more active than women (42.7% men; 27.9% women).

**Table 1:** Characteristics of the participants.

	Men	Women	*P*
	(*n* = 587)	(*n* = 613)	
Age (years)	51.0±19.0	50.7±19.0	0.766
Age groups (%)			
16–25 years old	10.7	11.1	0.841
26–45 years old	32.0	31.0	0.700
46–65 years old	29.3	29.9	0.834
≥66 years old	27.9	28.1	0.963
Weight (kg)	77.2±15.8	68.2±14.8	<0.001
Height (cm)	168.5±8.4	154.4±7.2	<0.001
BMI (kg/m^2^)	27.2±5.5	28.6±6.1	<0.001
BMI status (%)			
Underweight	2.2	1.6	0.461
Normal-weight	32.2	27.4	0.070
Overweight	42.1	34.9	0.011
Obesity	23.5	36.1	<0.001
WC (cm)	95.7±13.9	93.9±13.7	0.018
WHtR	0.57±0.09	0.61±0.09	<0.001
WHtR groups (%)			
<0.5	18.1	12.7	0.010
≥0.5–<0.6	52.1	34.7	<0.001
≥0.6	29.8	52.5	<0.001
Fasting glycaemia level	105.0±49.6	103.5±44.6	0.582
Treatment for diabetes (%)	3.1	4.1	0.346
HDL-c (mg/dL)	40.9±11.7	43.5±11.8	<0.001
Total cholesterol (mg/dL)	198.0±40.5	201.2±41.3	0.174
TG (mg/dL)	173.7±98.6	165.4±80.3	0.109
SBP (mmHg)	124.0±16.2	122.8±17.9	0.252
DBP (mmHg)	78.8±10.6	78.4±12.7	0.466
Treatment for hypertension (%)	6.6	6.2	0.753
Physical activity level (%)*			
Low	37.2	47.2	0.001
Moderate	20.2	24.9	0.077
High	42.7	27.9	<0.001

Abbreviations: SD, standard deviation; BMI, body mass index; WC, waist circumference; WHtR, waist-to-height ratio; HDL-c, high-density lipoprotein cholesterol; LDL-c, low-density lipoprotein cholesterol; VLDL-c, very low-density lipoprotein cholesterol; TG, triglyceride level; SBP, systolic blood pressure; DBP, diastolic blood pressure.

Values are presented as mean ± SD unless otherwise stated. Significant differences between men and women by unpaired *t*-test.

When values are expressed as %, significant differences between men and women were evaluated by χ^2^ test.

*The sample size for physical activity was n = 977, 471 men and 506 women.

Table 2 reports the prevalence of MetS components among adults (high fasting glycaemia, hypertriglyceridemia, low HDL-cholesterol, abdominal obesity and hypertension) according to gender, age and BMI status. The overall prevalence of MetS was 54.8%. This prevalence was significantly higher in women (60.4%) than in men (48.9%). Overall, there was not a linear increase in the prevalence of any MetS component except for BP with age. The criteria for MetS were met by 73.8% of obese adults (70.3% men; 76.0% women), 57.9% of overweight adults (55.5% men; 57.9% women) and by 32.9% of under/normal weight adults (26.2% men; 40.4% women). Abdominal obesity was the highest observed component of MetS (77.5%), while high fasting glycaemia (34.7%) was the lowest. When analysed by gender, abdominal obesity remained the highest component of MetS in both men and women. There were significant differences in HDL-cholesterol and abdominal obesity between men and women, whereas no significant differences between genders were reported in fasting glycaemia, hypertension and TGs.

**Table 2:** Prevalence (%) of metabolic syndrome components among adults in the State of Nuevo León, Mexico.

	High fastingglycaemia ordiabetic treatment	High TG	Low HDL-c	Abdominalobesity (WC)	Hypertension orantihypertensivetreatment	Metabolicsyndrome
Total (n = 1200)	34.7	48.9	61.3	77.5	39.4	54.8
Sex						
Men (n = 587)	35.8	48.6	47.9***	68.3***	40.7	48.9***
Women (n = 613)	33.6	49.3	74.2	86.3	38.2	60.4
Age and sex						
16–25 years old						
Total (n = 131)	12.2	28.2	53.4	45.0	14.5	22.1
Men (n = 63)	9.5	27.0	34.9***	30.2**	19.0	11.1**
Women (n = 68)	14.7	29.4	70.6	58.8	10.3	32.4
26–45 years old						
Total (n = 378)	26.7	47.4	65.3	77.0	22.0	49.7
Men (n = 188)	29.8	52.7*	50.5***	69.7**	20.7	47.3
Women (n = 190)	23.7	42.1	80.0	84.2	23.2	52.1
46–65 years old						
Total (n = 355)	45.4	60.3	63.7	85.6	48.5	66.2
Men (n = 172)	47.7	58.7	52.9***	76.2***	52.3	60.5*
Women (n = 183)	43.2	61.7	73.8	94.5	44.8	71.6
>65 years old						
Total (n = 336)	41.1	46.7	57.4	82.1	59.2	61.0
Men (n = 164)	40.2	41.5	44.5***	73.2***	59.8	53.0**
Women (n = 172)	41.9	51.7	69.8	90.7	58.7	68.6
BMI status and sex						
Under/normal-weight						
Total (n = 380)	23.9	35.5	51.8	45.5	32.6	32.9
Men (n = 202)	24.8	34.2	40.6***	32.2***	37.1*	26.2**
Women (n = 178)	23.0	37.1	64.6	60.7	27.5	40.4
Overweight						
Total (n = 461)	35.8	51.4	62.3	88.1	37.1	57.9
Men (n = 247)	37.2	53.8	50.6***	82.6***	37.2	55.5
Women (n = 214)	34.1	48.6	75.7	94.4	36.9	60.7
Obesity						
Total (n = 359)	44.6	59.9	70.2	97.8	49.6	73.8
Men (n = 138)	49.3	60.1	53.6***	95.7*	52.2	70.3
Women (n = 221)	41.6	59.7	80.5	99.1	48.0	76.0

Abbreviations: BMI, body mass index; TG, triglyceride level; HDL-c, high-density lipoprotein cholesterol; WC, waist circumference.

Significant differences between men and women by χ^2^ (*P<0.05, **P<0.01, ***P<0.001).

The association between MetS risk by each component (high fasting glycaemia, hypertriglyceridemia, low HDL-cholesterol, abdominal obesity and hypertension) and age was also assessed (Table 3). The risk of having abdominal obesity, low HDL-cholesterol level, hypertriglyceridemia, hypertension, and hyperglycaemia are higher among subjects with MetS at all studied ages. The univariate analysis of the total sample showed that the risk of cardiovascular risk factors were also associated with age in both men and women.

**Table 3:** Prevalence and odds ratio (95% confidence interval) among adults by age in the State of Nuevo León, Mexico.

	Total			Men			Women		
Cardiovascular risk factors	WithoutMetS†	WithMetS†	Crude OR(95% CI)‡	WithoutMetS†	WithMetS†	Crude OR(95% CI)‡	WithoutMetS†	WithMetS†	Crude OR(95% CI)‡
	(*n* = 543)	(*n* = 657)	(*n* = 977)	(*n* = 300)	(*n* = 287)	(*n* = 587)	(*n* = 243)	(*n* = 370)	(*n* = 613)
High fasting glycaemia									
Total	9.9	55.1***		13.3	59.2***		5.8	51.9***	
16–25 years old	5.9	34.5***	1.00 (ref.)	10.7	0.0	1.00 (ref.)	0.0	45.5***	1.00 (ref.)
26–45 years old	10.0	43.6***	2.62 (1.48–4.64)**	13.1	48.3***	4.03 (1.64–9.89)**	6.6	39.4***	1.80 (0.85–3.81)
46–65 years old	14.2	61.3***	5.97 (3.40–10.48)***	17.6	67.3***	8.66 (3.54–21.14)***	9.6	56.5***	4.41 (2.12–9.16)***
>65 years old	9.2	61.5***	5.01 (2.84–8.83)***	11.7	65.5***	6.40 (2.61–15.69)***	5.6	58.5***	4.18 (2.00–8.72)***
High TG									
Total	15.5	76.6***		20.3	78.0***		9.5	75.4***	
16–25 years old	15.7	72.4***	1.00 (ref.)	21.4	71.4*	1.00 (ref.)	8.7	72.7***	1.00 (ref.)
26–45 years old	15.3	79.8***	2.29 (1.49–3.52)***	22.2	86.5***	3.01 (1.61–5.63)**	7.7	73.7***	1.75 (0.96–3.17)
46–65 years old	21.7	80.0***	3.86 (2.49–5.96)***	26.5	79.8***	3.85 (2.04–7.25)***	15.4	80.2***	3.87 (2.13–7.07)***
>65 years old	9.9	70.2***	2.23 (1.44–3.45)***	11.7	67.8***	1.92 (1.01–3.63)*	7.4	72.0***	2.57 (1.41–4.70)**
Low HDL-c									
Total	34.6	83.4***		22.0	74.9***		50.2	90.0***	
16–25 years old	42.2	93.1***	1.00 (ref.)	26.8	100.0***	1.00 (ref.)	60.9	90.9*	1.00 (ref.)
26–45 years old	40.5	90.4***	1.64 (1.10–2.46)*	21.2	83.1***	1.90 (1.05–3.44)*	61.5	97.0***	1.67 (0.89–3.13)
46–65 years old	25.0	83.4***	1.53 (1.02–2.29)*	19.1	75.0***	2.09 (1.15–3.81)*	32.7	90.1***	1.17 (0.63–2.17)
>65 years old	29.0	75.6***	1.18 (0.78–1.77)	22.1	64.4***	1.50 (0.82–2.73)	38.9	83.9***	0.96 (0.52–1.78)
Abdominal obesity (WC)									
Total	57.6	93.9***		48.3	89.2***		69.1	97.6***	
16–25 years old	30.4	96.6***	1.00 (ref.)	21.4	100.0***	1.00 (ref.)	41.3	95.5***	1.00 (ref.)
26–45 years old	61.1	93.1***	4.08 (2.68–6.21)***	52.5	88.8***	5.32 (2.86–9.91)***	70.3	97.0***	3.73 (2.01–6.95)***
46–65 years old	67.5	94.9***	7.27 (4.62–11.46)***	55.9	89.4***	7.40 (3.89–14.06)***	82.7	99.2***	12.11 (5.44–26.94)***
>65 years old	64.9	93.2***	5.61 (3.60–8.74)***	55.8	88.5***	6.32 (3.33–11.97)***	77.8	96.6***	6.83 (3.37–13.82)***
Hypertension									
Total	19.5	55.9***		25.0	57.1***		12.8	54.9***	
16–25 years old	7.8	37.9***	1.00 (ref.)	14.3	57.1*	1.00 (ref.)	0.0	31.8***	1.00 (ref.)
26–45 years old	5.8	38.3***	1.66 (0.96–2.86)	9.1	33.7***	1.11 (0.54–2.29)	2.2	42.4***	2.63 (1.12–6.16)*
46–65 years old	28.3	58.7***	5.54 (3.26–9.40)***	35.3	63.5***	4.67 (2.33–9.36)***	19.2	55.0***	7.08 (3.07–16.30)***
>65 years old	40.5	71.2***	8.56 (5.03–14.59)***	44.2	73.6***	6.31 (3.13–12.73)***	35.2	69.5***	12.40 (5.36–28.69)***

Abbreviations: MetS, metabolic syndrome; OR, odds ratio; CI, confidence interval; TG, triglyceride level; HDL-c, high-density lipoprotein cholesterol; WC, waist circumference.

† Values are %. Statistical analysis was performed by χ^2^ and two-tailed Fisher’s exact test when the expected frequency in any cell was less than 5.

‡ Univariate analysis of the total sample (logistic regression analysis considering the effect of one explanatory variable) was used to assess the association between age (independent variable) and each of the MetS component (dependent variables).

*P<0.05, **P<0.01, ***P<0.001.

The association of anthropometric variables (weight, height, BMI, WC and WHtR) with MetS is reported in Table 4. Men and women having MetS showed significantly greater values for all anthropometric variables analysed with the exception of height.

**Table 4:** Anthropometric characteristics with or without a diagnosis of metabolic syndrome among adults in the State of Nuevo León, Mexico.

	Men			Women		
	WithoutMetS	WithMetS	*P*	WithoutMetS	WithMetS	*P*
	(*n* = 300)	(*n* = 287)		(*n* = 243)	(*n* = 370)	
Weight (kg)	72.3±15.1 (70.6–74.0)	82.4±15.1 (80.6–84.1)	<0.001	62.9±14.3 (61.1–64.7)	71.7±14.2 (70.3–73.2)	<0.001
Height (cm)	168.0±8.3 (167.0–168.9)	169.1±8.3 (168.2–170.1)	0.100	154.5±6.8 (153.6–155.3)	154.4±6.8 (153.7–155.1)	0.974
BMI (kg/m^2^)	25.7±5.4 (25.1–26.3)	28.8±5.4 (28.2–29.4)	<0.001	26.4±5.9 (25.7–27.2)	30.0±5.9 (29.4–30.6)	<0.001
WC (cm)	91.1±12.6 (89.6–92.5)	100.7±12.7 (99.2–102.1)	<0.001	88.2±12. (86.6–89.8)	97.6±12.7 (96.3–98.9)	<0.001
WHtR	0.54±0.1 (0.54–0.55)	0.60±0.1 (0.59–0.61)	<0.001	0.57±0.1 (0.56–0.58)	0.63±0.1 (0.62–0.64)	<0.001

Abbreviations: MetS, metabolic syndrome; BMI, body mass index; WC, waist circumference; WHtR, waist-to-height ratio. Values are mean ± SD (95% confidence interval). Statistical analysis was performed by ANCOVA adjusted by age.

Table 5 shows the relationship between MetS components and the physical activity level of Mexican adults. Low prevalence of MetS was found in the high physical activity category in men. However, logistic regression analysis showed no significant relation between MetS components and physical activity levels after adjustment by sex and age.

**Table 5:** Metabolic syndrome components risk (odds ratio, 95% confidence interval) by physical activity level among adults in the State of Nuevo León, Mexico.

	Physical activity level		
	Low	Moderate	High
*n* (men/women)	175/239	95/126	201/141
Metabolic syndrome (%)†			
Total	59.9	60.2	49.4**
Men	53.7	62.1	43.3**
Women	64.4	58.7	58.2
Metabolic syndrome criteria (OR, 95%CI)‡§			
Glucose (≥100 mg/dL) or diabetic treatment			
Total^§^	1	0.91 (0.65–1.28)	0.82 (0.60–1.11)
Men^‡^	1	0.88 (0.52–1.48)	0.83 (0.53–1.27)
Women^‡^	1	0.92 (0.58–1.45)	0.77 (0.49–1.20)
Triglycerides (≥150 mg/dL)			
Total^§^	1	0.89 (0.64–1.24)	0.86 (0.64–1.16)
Men^‡^	1	0.92 (0.56–1.52)	0.86 (0.56–1.30)
Women^‡^	1	0.84 (0.54–1.30)	0.79 (0.52–1.20)
HDL-cholesterol (<40/50 mg/dL men/women)			
Total^§^	1	0.99 (0.70–1.41)	0.86 (0.63–1.17)
Men^‡^	1	1.43 (0.86–2.37)	1.09 (0.72–1.67)
Women^‡^	1	0.72 (0.44–1.17)	0.68 (0.43–1.09)
Waist circumference (≥102/88 cm men/women)			
Total^§^	1	0.79 (0.52–1.20)	0.83 (0.57–1.20)
Men^‡^	1	0.81 (0.46–1.44)	0.64 (0.40–1.03)
Women^‡^	1	0.75 (0.41–1.38)	1.44 (0.73–2.83)
Hypertension (≥130/85 mmHg) or antihypertensive treatment			
Total^§^	1	1.25 (0.87–1.78)	1.11 (0.81–1.53)
Men^‡^	1	1.64 (0.95–2.82)	1.21 (0.76–1.92)
Women^‡^	1	1.01 (0.63–1.62)	1.07 (0.68–1.68)
Metabolic syndrome (≥3 criteria)			
Total^§^	1	1.08 (0.77–1.52)	0.77 (0.57–1.04)
Men^‡^	1	1.57 (0.94–2.65)	0.79 (0.51–1.20)
Women^‡^	1	0.80 (0.51–1.25)	0.77 (0.50–1.19)

† Values are %.

‡§ Multivariate analyses (multiple logistic regressions considering the simultaneous effect of each explanatory variable adjusted for ^‡^age and ^§^sex were used to assess the association between physical activity level (independent variable) and each of the metabolic syndrome components (dependent variables).

## Discussion

The main finding of this study is that, at present, MetS prevalence in adults aged ≥16 living in the State of Nuevo León, Mexico, is 54.9% (45.3% men and 59.4% women) using the consensus definition (IDF/NHLBI/AHA/WHF/IAS/IASO) [14]. These findings prove that MetS prevalence has been progressively increasing in Mexico since the first nutritional surveys, 26.6% in 1992–1993 [7], 34% in 2000 [16], 36.8% in 2006 [8], and 45% in 2012 [9]. These previous studies used different definitions of MetS (ATP-III, NHLBI/AHA, and IDF) and age ranges respect to the present study. Nevertheless, despite differences in studied ages and the definition of MetS these studies indicate that the prevalence of MetS is progressively increasing.

A recent systematic review previously reported that the prevalence of MetS in Latin-American countries ranges from 18.8% to 43.3% [17]. These values for prevalence of MetS in Latin America are similar to the ranges obtained for adult people in Europe and USA [18]–[20]. Therefore, Mexico shows a considerably higher prevalence of MetS than the nearest countries, such as El Salvador (28.8%) [21], Guatemala (22.7% in men, and 41.1% in women) [22] and Costa Rica (29.2%) [23], and present findings from the State of Nuevo León also show results above this range. The cause of this trend may be related to changes in lifestyle, characterized by a higher energy intake and low physical activity, as well as by massive migrations from rural to urban areas [24].

MetS is a combination of risk factors, including abdominal obesity, hyperglycaemia, hypertriglyceridemia, hypertension, and low HDL-cholesterol, which significantly increase the risk of developing cardiovascular diseases, and type 2 diabetes mellitus. The present findings clearly demonstrate that MetS is more prevalent among obese adults (73.7%) when compared with overweight (58.0%) and normal weight adults (36.7%). A significant prevalence was observed for all components of the syndrome, which were higher for the obese with respect to overweight and normal weight people from Nuevo León, with similar percentages to those reported in a previous study analysing cardiovascular risk factors in a Mexican urban population (The Lindavista Study) [25]. The prevalence of abdominal obesity was evidenced by the elevated number of obese or overweight people, reaching 97.5% in the obese and 83.5% in overweight adults. Abdominal obesity is associated with insulin resistance, hyperinsulinaemia, and increased risk of type 2 diabetes and vascular problems [26]. An interesting parameter to highlight was the high incidence of low HDL-cholesterol, which was also previously observed among Mexican population [25]. Moreover, more than half of the subjects had elevated triglyceride levels, a fact that might be inversely associated with HDL-cholesterol, which has been previously described [27].

The prevalence of MetS in Nuevo León was significantly higher in women than in men. The prevalence of MetS was also higher in women in a study analyzing the prevalence of MetS in an urban sample from Mexico City performed in 2011 [11]. Women were more affected than men due to the higher prevalence of central obesity and low HDL-cholesterol. These results are in accordance with the results obtained by the ENSANUT [8], [28]. Moreover, similar results were obtained in Mexican Americans with a MetS prevalence of 39.4% in men and 45.4% in women [29]. In a previous study, it was found that socioeconomic position, measured by education and income, is associated with MetS in Mexican women, which could partially explain the present results [30]. Future research focused on identifying mechanisms responsible for gender differences in MetS prevalence will provide better knowledge about MetS possible determinants.

Previous epidemiological studies have demonstrated that daily physical activity prevents both the incidence of chronic diseases such as MetS and premature death [31]. Physical activity which involves higher energy expenditures and increases physical fitness has demonstrated to decrease the risk of MetS [31]–[33]. Overall, the current results reported a decrease in the percentage of subjects with MetS with increasing physical activity levels; however, there was not a clear relationship between MetS and its components and the degree of physical activity when performing a multivariate analysis controlled by sex and age. Although active subjects are less likely to suffer from MetS, these results are not significant compared to the sedentary group, suggesting that a main role is played by other factors, such as dietary pattern [34]–[35]. Accordingly, it was reported that fat mass, rather than inactivity, is an important contributor to disease risk in Mexican and Mexican–American women living on the US/Mexico border [36].

The dietary habits of the Mexican population have changed dramatically over the last few years, and this is reflected in an increased prevalence of overweight and obesity [37]. Consumption of a Western dietary pattern, characterized by a high intake of pastries, refined cereals, corn tortillas, and soft drinks and low consumption of whole cereals, seafood, and dairy products, was associated with the components of MetS in Mexican adults, by providing excess energy and large amounts of rapidly absorbable sugars [38]. In addition, increased availability of inexpensive energy-dense foods in rural and urban areas has been reported [39]. Given the high prevalence of MetS and obesity in the Mexican population, and the lack of relation to physical activity, more research is essential in order to find the factors involved in the establishment of MetS among the Mexican adult population.

## Conclusions

The present results report that more than 50% of adult individuals of Nuevo León (Mexico) suffer from metabolic syndrome. Obese adults, mainly women, are particularly at risk of developing MetS, with significant implications for their health, mainly cardiovascular disease and diabetes. These results highlight the importance of weight loss to reduce morbidities associated with MetS. However, no direct relation was evidenced between physical exercise and MetS prevalence, indicating that there are other causes that make a greater contribution to this syndrome. Altogether, the subject demands more research and preventive and therapeutic strategies focussed on diet, exercise and lifestyle changes.

## Strengths and Limitations

The main strength of the present study includes a large population-based sample (*n* = 1200) that provides greater support for generalisation. This is the first study to use the joint consensus definition of the MetS among Mexican population. An additional strength is confirmation of the increasing trend in MetS prevalence among the Mexican population, making it necessary to develop effective preventive measures. However, this article also has several limitations. First, the cross-sectional study design reduced the ability to show causality compared with longitudinal studies. Second, questionnaires have inherent limitations, mainly because they are subjective in nature, and also there is not information about socioeconomic status. Self-report of physical activity can lead to over-report the physical activity, on a social desirability bias, and therefore the number of inactive individuals may be lower than that reported, especially among the obese. Finally, data on the use of lipid-lowering medication is not available in EESN-NL 2011/2012, therefore treatment with lipid-lowering medication was not considered as a positive variable despite that has a substantial impact on all lipid parameters and this may underestimated the prevalence of participants having altered serum lipid levels.
